# Current understanding on inferior quality of liver grafts by donation after circulatory death based on multi-omics data

**DOI:** 10.3389/fimmu.2025.1548735

**Published:** 2025-03-20

**Authors:** Yifeng Zhou, Ting Que, Lu Yu, Shuping Que, Jun Xu, Zhengtao Liu

**Affiliations:** ^1^ Key Laboratory of Artificial Organs and Computational Medicine in Zhejiang Province, Shulan International Medical College, Zhejiang Shuren University, Hangzhou, China; ^2^ Birth Defects Prevention and Control Institute, Maternal and Child Health Hospital of Guangxi Zhuang Autonomous Region, Nanning, China; ^3^ School of Medicine, Zhejiang Chinese Medical University, Hangzhou, China; ^4^ Shulan (Hangzhou) Hospital, Hangzhou, China; ^5^ Ya-er-zhuang Clinics, Hangzhou, China; ^6^ Division of Hepatobiliary and Pancreatic Surgery, Department of Surgery, First Affiliated Hospital, School of Medicine, Zhejiang University, Hangzhou, China; ^7^ NHC Key Laboratory of Combined Multi-organ Transplantation, Key Laboratory of the Diagnosis and Treatment of Organ Transplantation, School of Medicine, Chinese Academy of Medical Sciences, First Affiliated Hospital, Zhejiang University, Hangzhou, China; ^8^ Key Laboratory of Organ Transplantation, First Affiliated Hospital, School of Medicine, Zhejiang University, Hangzhou, China

**Keywords:** donation after circulatory death, liver transplantation, multi-omics, ischemia-reperfusion injury, oxidative stress, inflammatory response

## Abstract

Given the inevitable hypoxia and reperfusion injury that occur in organs donated after circulatory death (DCD), the quality and function of these organs are significantly compromised, greatly limiting their application in clinical organ transplantation. Recently, the advancement of functional omics technologies has enabled us to deeply analyze the mechanisms underlying DCD donor organ damage from multiple perspectives. This review systematically integrates the studies from transcriptomics, proteomics, and metabolomics to reveal the key biological mechanisms associated with the declines in DCD donor organ quality, including oxidative stress, inflammatory responses, cell death pathways, and metabolic disturbances. Additionally, we summarized emerging therapeutic strategies based on findings from omics perspectives, offering new possibilities to improve the quality of DCD organ for better transplant prognosis. Finally, we discussed the challenges in current research and future directions to provide scientific evidence for clinical practice and promote the application of DCD donors in organ transplantation.

## 1 Introduction

The rapid growth in global demand for organ transplantation has made prominent donor shortages. Traditional donation after brain death (DBD) has limited the organ application which can’t meet the urgent need for transplantable organs. To alleviate the contradictions between organ supply and demand for transplantation, organs from donation after circulatory death (DCD) has become an important alternative source. Unlike DBD donors, DCD donors experience a prolonged period of no blood flow after circulatory arrest, leading to severe ischemic damage before transplantation and further injury after reperfusion (1). Series of injuries significantly affects the quality of DCD grafts, limiting their application in clinical practice.

Ischemia-reperfusion injury (IRI) is key factor to cause poor quality DCD organs. The IRI process involves multiple complex biological events including oxidative stress, intracellular calcium overload, inflammatory responses, and apoptosis (2). These events cause extensive cellular damage, indicating more organ’s functional instability and risk of acute/chronic post-transplant failure. Despite various interventions including hypothermic preservation and machine perfusion with aims to reduce the impact of IRI, DCD still limits the use for organ transplantation for inferior posttransplant prognosis (3). In-depth research into the molecular mechanisms of DCD donor organ injury and the exploration of more effective protective strategies have become the focus of current studies.

The development of omics technologies provides new perspectives for understanding the mechanisms of IRI in DCD donor organs. Omics encompasses a variety of techniques, including genomics, transcriptomics, proteomics, and metabolomics, which can systematically reveal the biological changes in cells and tissues under stress conditions. For example, genomic techniques can identify gene mutations or polymorphisms associated with IRI, helping to determine susceptibility markers. Transcriptomics can analyze dynamic changes in gene expression at different time points, revealing the activation or inhibition of specific signaling pathways (4). Proteomics not only quantifies protein expression levels but also detects protein modifications, uncovering key regulatory mechanisms during IRI. Metabolomics, by monitoring changes in metabolites, provides a deeper understanding of cellular metabolic disorders.

In DCD donor research, these functional omics techniques have been widely applied in various aspects. For instance, transcriptomic analysis has identified multiple inflammatory factors and signaling pathways related to IRI, offering potential targets for anti-inflammatory therapies. Additionally, proteomics studies have revealed the central role of oxidative stress in DCD donor organ injury, prompting researchers to explore the application of antioxidants in organ protection. Metabolomics, by detecting changes in energy metabolism, helps to understand the mechanisms of mitochondrial dysfunction during ischemia-reperfusion. These research findings not only deepen our understanding of the mechanisms of DCD donor organ injury but also provide new approaches for clinical interventions.

Despite significant progress in studying the mechanisms of DCD donor organ injury through functional omics, many issues remain unresolved. First, the integration and collaborative research between different functional omics techniques have not been fully developed. While single-omics studies can provide abundant data, the understanding of complex biological processes remains limited. Through multi-omics integration, a more comprehensive analysis of the global changes occurring in DCD donor organs during ischemia-reperfusion can be achieved, revealing more complex molecular mechanisms (5). Second, the interpretation of functional omics data and its clinical translation still face challenges. The vast amount of omics data requires complex bioinformatics methods for analysis and interpretation, and how these results can be translated into actionable clinical interventions is a problem that needs to be addressed. Additionally, the time and cost requirements of functional omics research are high, limiting its application in large-scale clinical studies.

Against this backdrop, future research needs to further explore methods for integrating multi-omics approaches to systematically analyze the complex mechanisms of DCD donor organ injury. At the same time, efforts should be made to strengthen the connection between functional omics data and clinical practice, promoting the clinical translation of functional omics research through interdisciplinary collaboration. These efforts will help develop more effective protective strategies for DCD donor organs, improve the success rate of organ transplants, and ultimately enhance the survival quality of patients.

Given the significant impact of DCD on liver quality and transplant outcomes, and the key insights provided by omics data, our study aimed to explore the molecular mechanisms underlying these effects. We focus on key biological pathways leading to liver injury in DCD, such as oxidative stress, inflammation, and mitochondrial dysfunction. Additionally, we identified potential therapeutic targets through omics studies that could provide valuable intervention points to improve.

## 2 Impact of DCD on inferior post-operative prognosis

Donation after circulatory death (DCD) refers to the process of organ donation that occurs after the patient’s heart has ceased beating and irreversible death has been precisely confirmed (6). The widespread adoption of organ donation has necessitated the use of DCD liver transplantation This donation model supplements traditional donation after brain death (DBD) and helps alleviate the critical global shortage of organs. DCD donation gained renewed attention in the 1990s, particularly in Europe and the United States, where it has gradually become an important source for organ transplantation (7).

The potential sources of DCD donors primarily include patients who are deemed unlikely to recover from severe brain injury or other conditions leading to irreversible deterioration, where life support is withdrawn at the patient’s or family’s decision (8). Once life support is withdrawn, the patient’s heart will cease activity within a predetermined period, at which point death is declared. After confirming circulatory death according to relevant standards, the organ retrieval process can begin.

However, the acquisition and utilization of DCD donor organs face significant challenges. Due to the period of oxygen deprivation following cardiac arrest, DCD donor organs often suffer from severe ischemia-reperfusion injury (IRI) (9). This damage affects the quality of the organs and can lead to post-transplant dysfunction or even transplant failure. In the process of liver injury after Donation after Circulatory Death (DCD), oxidative stress and mitochondrial dysfunction are key factors contributing to the decline in liver function and poor post-transplant prognosis. During ischemia-reperfusion, reactive oxygen species (ROS) are significantly elevated, leading to mitochondrial membrane damage, which impairs hepatic cell energy supply and metabolic functions. These oxidative damages not only damage the liver’s basic physiological functions but also increase the risk of apoptosis and necrosis (10). Markers of oxidative stress, such as hydrogen peroxide (H2O2) and malondialdehyde (MDA), are significantly elevated in DCD livers, indicating severe oxidative damage during ischemia-reperfusion (2). Additionally, the downregulation of antioxidant enzymes like superoxide dismutase (SOD) and glutathione peroxidase (GPX) reduces the liver’s resistance to oxidative damage, further exacerbating the injury (11). Simultaneously, mitochondrial dysfunction plays a crucial role in DCD liver injury. In DCD livers, key proteins involved in the mitochondrial electron transport chain (ETC), such as NDUFS3 and ATP5A1, are significantly downregulated, leading to reduced ATP synthesis and impaired liver energy metabolism (5). The oxidative stress-induced lipid peroxidation and metabolic disturbances (such as succinate accumulation and the elevation of NADH/NAD+ ratio) further aggravate mitochondrial dysfunction, impairing liver recovery after transplantation (12). Thus, oxidative stress and mitochondrial dysfunction play critical roles in DCD liver injury and poor post-transplant outcomes, with profound effects on liver function recovery following transplantation. As a result, the success rate of DCD organ transplants is generally lower than that of DBD donor organs.

To address these challenges, various techniques and strategies have been developed to improve the quality of DCD donor organs. For example, enhanced mechanical perfusion techniques can restore blood supply to the organs immediately after cardiac arrest, thereby reducing ischemic time and minimizing organ damage (13). Additionally, the application of functional omics provides new insights into the molecular changes occurring in DCD donor organs during reperfusion. These research findings offer scientific evidence for optimizing the preservation and transplantation strategies of DCD organs, promoting their broader clinical application (14).

Nevertheless, the use of DCD donor organs still faces many technical and ethical challenges. How to maximize the utilization of donor organs while ensuring their quality, and how to adhere to strict ethical standards during organ retrieval, are issues that require further exploration in the future (15). With advancements in technology and ongoing research, the success rate of DCD organ transplantation is expected to improve, offering more opportunities for patients in need of organ transplants.

## 3 Current status of functional omics research on cardiac death donation donors

To provide a comprehensive overview of the current literature on clinical omics studies related to DCD liver transplantation, **Table 1** summarizes key features of original reports, including study comparisons, donor demographics, primary causes of liver transplantation, sample types, assay methods, key molecules, pathways, and major findings. These studies highlight the diverse approaches and significant discoveries in the field, offering insights into the molecular mechanisms underlying the inferior quality of DCD liver grafts.

**Table 1:** Literature features with original reports of clinical omics for DCD.

Author, Country, Publication year [Reference]	Comparison	Donor Gender Ratio (Male/Female)	Primary Cause of Liver Transplan	Sample	Assay	Key molecule	Key pathway	Major finding
Olga Hrydziuszko, UK, 2016 (16)	DCD vs DBD	DCD:2/8 DBD:11/16	ALD:14 PSC:6 PBC:5 PCLD:2 HCV:6	Liver tissue, Preoperatively	Metabolomics	1. Tryptophan 2. Kynurenine 3. Glutathione 4. SAM	1. Purine Metabolism 2. Tryptophan Metabolism 3. Oxidative Phosphorylation 4. TCA Cycle	1. Higher levels of tryptophan and kynurenine in DCD livers, which might be linked to the higher rate of graft failure in DCD donors. 2. These metabolites could serve as important biomarkers to help predict post-transplant outcomes.
Jin Xu, UK, 2015 (17)	DCD vs DBD	DCD:9/9 DBD:16/22	ALD:10 PSC:6 HCV:1 HCC:1	Liver tissue, Preoperatively	Metabolomics	1. LysoPC (16:0), 2. LysoPC (18:0)	1. PC 2. LysoPC 3. PAF	1. Lipid Differences: Higher levels of lysophospholipids (LysoPC 16:0 and LysoPC 18:0) were found in DCD liver grafts compared to DBD. 2. Biomarkers for Graft Quality: These lysophospholipids are linked to increased liver dysfunction and could serve as biomarkers for early allograft dysfunction. 3. Predictive Value: Lysophospholipid levels showed an 82% accuracy in predicting early allograft dysfunction.
Xinguo Chen, CHN, 2018 (18)	IFvsSDGFvsLDGF	NA	NA	Liver tissue, Preoperatively	Transcriptome	1. Nup62 2. Hsd17b10 3. RNApolymerase II 5. NPM1	1. Cell cycle	1. The dynamic changes occurring in DCD livers over time, which may affect their suitability for transplantation. 2. Nup62 and Hsd17b10 were identified, which are important for nuclear production exchange and liver metabolism, respectively.
Thamara Prabath Ranasinghe Perera, UK, 2015 (19)	DCD vs DBD	DCD:4/9 DBD:11/16	ALD:4 PSC:4 PBC:5 PCLD:2 HCV:6 NASH:1	Liver tissue, Preoperatively	Metabolomics	1. Tryptophan 2. Kynurenine	1. Tryptophan metabolism	1. In liver transplant donors donated after circulatory death (DCD), tryptophan and kynurenine levels were significantly associated with those in donors donated after brain death (DBD). 2. Increases in these metabolites may be associated with lack of blood damage in DCD donors and may predict potential biomarkers of liver transplant function

ALD, Alcoholic Liver Disease; HCC, Hepatocellular Carcinoma; HCV, Hepatitis C Virus; IF, Immuno fluorescence; LDGF, Long Duration Graft Dysfunction; LysoPC, Lysophosphatidylcholine; NASH, Non-alcoholic steatohepatitis; PAF, Platelet-activating factor; PC, Phosphatidylcholine; PCLD, Polycystic Liver Disease; PSC, Primary Sclerosing Cholangitis; SAM, S-Adenosylmethionine; SDGF, Short Duration Graft Dysfunction.

### 3.1 Overview of functional omics approaches

One of the major differences between cardiac death donors (DCD) and brain death donors (DBD) is the severity of ischemia-reperfusion injury. Studies show that DCD livers experience more significant ischemic damage due to prolonged cessation of blood flow. Compared to DBD livers, DCD livers exhibit more severe oxidative stress, inflammatory cytokine release, and mitochondrial dysfunction during ischemia-reperfusion. Therefore, understanding these biological differences between DCD and DBD donors is critical for developing effective protective strategies.”Functional omics, including genomics, transcriptomics, proteomics, and metabolomics, is crucial in understanding complex biological changes in DCD organs. These approaches identify molecular pathways linked to ischemia-reperfusion injury (IRI) and other damage types, guiding the development of targeted interventions.

### 3.2 Transcriptomics: analyzing gene expression

Transcriptomic analyses of DCD liver grafts reveal dynamic alterations in gene expression patterns, particularly in inflammatory, apoptotic, and metabolic pathways. A significant upregulation of IL-6, TNF-α, and CXCL8 (IL-8) mRNA has been observed in DCD livers, correlating with prolonged leukocyte infiltration and endothelial activation (10). Additionally, the expression of HIF-1α and its downstream targets (e.g., VEGFA, GLUT1) is markedly elevated, indicating an adaptive response to ischemic stress (20). Furthermore, transcriptome-wide analyses highlight a suppression of key mitochondrial biogenesis regulators, including PGC-1α and NRF1, suggesting an impaired mitochondrial recovery post-reperfusion (2). These findings provide insights into the transcriptional landscape governing DCD graft injury and potential therapeutic targets for mitigating ischemia-induced damage.

### 3.3 Proteomics: uncovering protein modifications

Proteomic studies have characterized the post-ischemic protein landscape in DCD liver grafts, identifying a marked decline in mitochondrial oxidative phosphorylation (OXPHOS) complex proteins, particularly NDUFS3 and SDHB, components of complex I and II, respectively (2). The attenuation of these electron transport chain (ETC) proteins correlates with reduced ATP synthesis and an accumulation of succinate, a hallmark of ischemia-driven metabolic dysregulation (12). Concurrently, proteomic profiling has uncovered increased carbonylation and nitration of mitochondrial proteins, such as ATP5A1 and SOD2, reflecting oxidative modifications that impair enzymatic activity (21). Notably, HSP90, a key chaperone involved in protein quality control, is upregulated, suggesting an adaptive stress response to misfolded protein accumulation in ischemic grafts (5). These protein-level alterations indicate that mitochondrial dysfunction in DCD livers is not only a consequence of ischemia but also driven by extensive post-translational modifications (PTMs) affecting OXPHOS efficiency and antioxidant capacity.

### 3.4 Metabolomics: insights into metabolic dysregulation

Metabolomic analyses of DCD livers have identified distinct metabolic signatures that differentiate ischemia-reperfusion injury (IRI)-affected grafts from non-IRI controls. One of the most striking findings is the accumulation of succinate and fumarate in DCD grafts, consistent with reverse electron transport (RET)-induced mitochondrial dysfunction (10). This accumulation is accompanied by an elevated NADH/NAD+ ratio, reflecting an impaired oxidative metabolism (5).

Additionally, a reduction in acylcarnitines (e.g., palmitoylcarnitine, stearoylcarnitine) has been observed, suggesting compromised fatty acid oxidation (FAO) in ischemic hepatocytes. This aligns with proteomic findings indicating a downregulation of carnitine palmitoyltransferase 1A (CPT1A), the rate-limiting enzyme in mitochondrial FAO (12).

Moreover, metabolomic profiling reveals an increased lactate/pyruvate ratio, indicative of a shift toward anaerobic glycolysis under ischemic conditions (22). The excess lactate accumulation contributes to intracellular acidosis, further exacerbating hepatocyte injury. Collectively, these metabolic disruptions highlight the profound impairment of mitochondrial energy metabolism in DCD livers and provide potential metabolic targets for therapeutic intervention.

## 4 Differences in functional omics between DCD and DBD livers

Previous studies have shown that DCD livers differ significantly from DBD livers in terms of functional status, metabolic capacity, and cellular homeostasis before and after transplantation. Functional omics approaches—including transcriptomics, proteomics, metabolomics, and multi-omics integration—provide a systematic understanding of these molecular differences, offering crucial insights for assessing and improving the quality of DCD donor livers.

Multiple studies have demonstrated that, following ischemia-reperfusion injury (IRI), genes related to inflammation and apoptosis are significantly upregulated in DCD livers compared to DBD livers (12, 23). For instance, pro-inflammatory cytokines IL-6 and TNF-α show increased mRNA expression in DCD livers, leading to a heightened immune response and tissue damage. Additionally, HIF-1α, a key regulator of cellular adaptation to hypoxia, exhibits dynamic changes in DCD livers, though its protective or detrimental role remains to be fully elucidated (24). Proteomic studies have revealed significant differences in protein expression and post-translational modifications between DCD and DBD livers. The expression of mitochondrial function-related proteins, such as ATP synthase, is significantly downregulated in DCD livers, suggesting impaired energy metabolism. Furthermore, antioxidant proteins such as superoxide dismutase (SOD) and glutathione peroxidase (GPX) are expressed at lower levels, making DCD livers more susceptible to oxidative stress damage (23). These findings suggest that protein expression patterns in DCD livers may influence postoperative recovery and increase the risk of transplant failure. Metabolomic studies indicate that metabolic homeostasis is severely disrupted in DCD livers. Key features include the accumulation of lactate and fatty acid oxidation products, reflecting metabolic acidosis and energy metabolism disorders (12). In addition, abnormal levels of glutamine and other amino acids in DCD livers may be associated with delayed recovery post-transplantation (24). These metabolic alterations further support the notion that DCD livers exhibit impaired functional recovery after transplantation. Recent multi-omics studies integrating transcriptomic, proteomic, and metabolomic data have provided deeper insights into the molecular characteristics of DCD livers. These studies have identified oxidative stress, inflammatory responses, and mitochondrial dysfunction as key factors contributing to DCD liver damage (12, 23, 24). The interplay of these biological pathways exacerbates the extent of injury in DCD livers and may be a primary mechanism underlying their poorer transplant prognosis.

**Figure 1:**
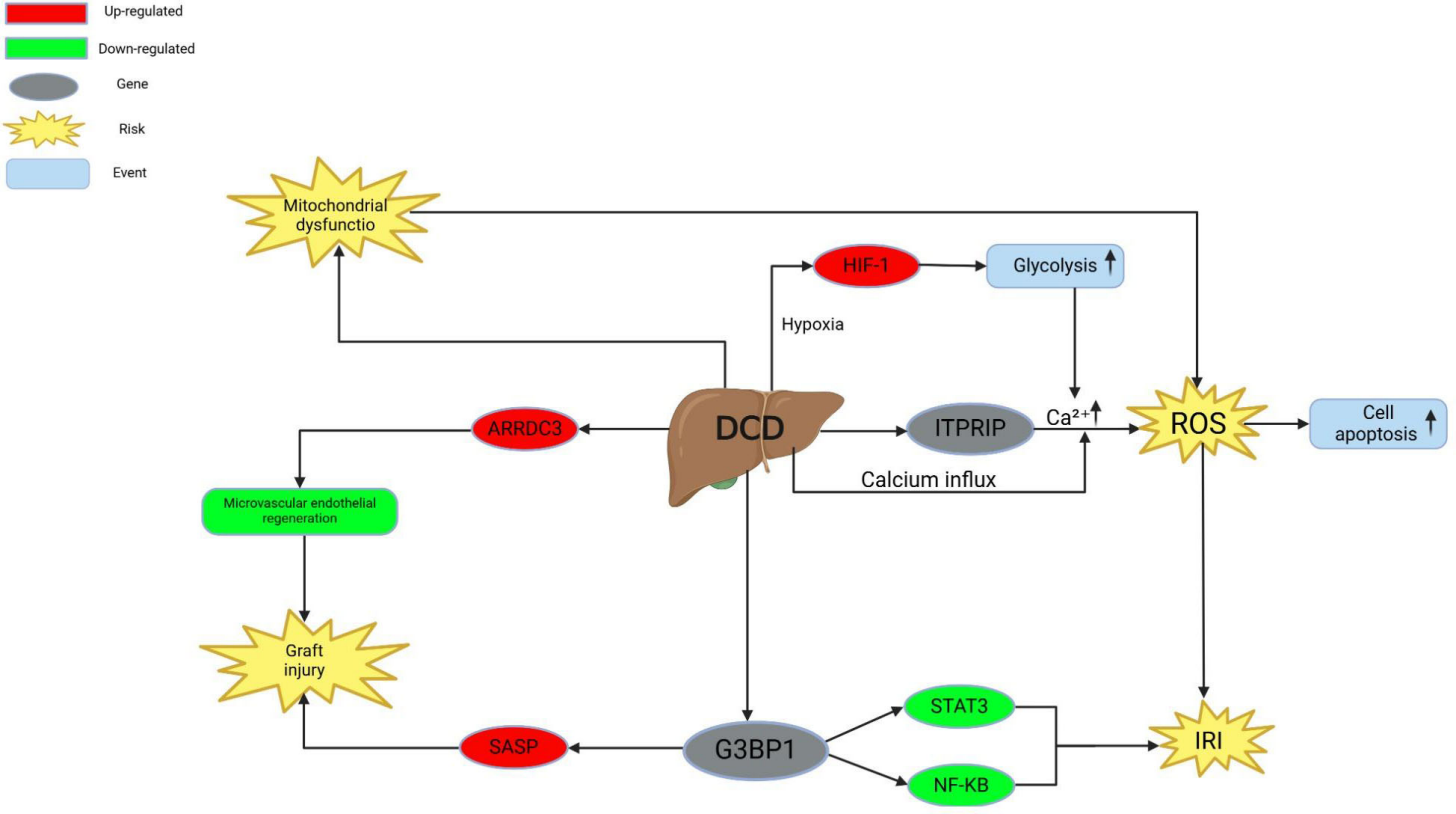
This figure illustrates the key molecular mechanisms by which DCD (Donation after Circulatory Death) leads to ischemia-reperfusion injury (IRI) during liver transplantation. It highlights the roles of HIF-1, ITPRIP, ARRD3, SASP, G3BP1, STAT3, and NF-KB in regulating graft injury, oxidative stress, and inflammation. ARRDC3, Asc-Amyloid Pyrin Domain Containing 3; DCD, Donation after Circulatory Death; G3BP1, GTPase 3-binding protein 1; HIF-1, Hypoxia Inducible Factor 1; ITPRIP, Intraperitoneal Triglycerin Infusion Period; IRI, Ischemia-Reperfusion Injury; NF-KB, Nuclear Factor kappa-light-chain-enhancer of B cells; ROS, Reactive Oxygen Species; SASP, Apoptosis-associated speck-like protein; STAT3, Signal Transducer and Activator of Transcription 3.

Collectively, previous studies indicate that DCD and DBD livers exhibit significant differences in inflammation, metabolism, antioxidant capacity, and mitochondrial function. Functional omics analyses provide essential molecular evidence explaining the increased risk of transplant failure in DCD livers and offer potential directions for improving transplantation strategies. Future research should focus on further integrating multi-omics data to unravel the complex mechanisms of DCD liver injury and explore more effective intervention strategies. **Figure 1** summarizes the integrated analysis results of transcriptomics, proteomics, and metabolomics, illustrating the key molecular characteristics of DCD livers in terms of oxidative stress, inflammatory responses, and mitochondrial function. This figure helps us understand how the complex molecular networks revealed by multi-omics integration contribute to the exacerbation of damage in DCD livers.

## 5 Emerging therapeutic options based on omics studies

With the rapid advancement of functional omics technologies, scientists have proposed a series of emerging therapeutic strategies by deeply analyzing the molecular mechanisms of ischemia-reperfusion injury (IRI) in DCD (donation after circulatory death) livers. These strategies include drug interventions, gene therapy, and metabolic regulation, all aimed at improving the function of DCD livers and increasing their transplantation success rates. One of the most widely researched and applied new therapeutic strategies based on functional omics studies is drug intervention. To further illustrate the therapeutic strategies for DCD liver grafts based on omics data, **Table 2** summarizes key studies that have explored various omics platforms to identify potential therapeutic targets and interventions. These studies highlight the diverse approaches and significant findings in the field, offering insights into the development of novel therapeutic strategies to improve the quality and function of DCD liver grafts. Through transcriptomic, proteomic, and metabolomic research, scientists have identified key molecules and pathways associated with DCD liver injury and developed various drugs targeting these factors. For example, studies have shown that oxidative stress response is significantly enhanced in DCD livers, leading to cellular damage and functional decline (11). In response to this finding, antioxidant drugs such as N-acetylcysteine (NAC) and vitamin E have been used in experimental studies, demonstrating the ability to reduce oxidative damage and protect mitochondrial function (21, 28). Additionally, based on proteomic research, drugs that inhibit inflammatory factors like TNF-α have been proposed to reduce inflammatory responses in DCD livers and improve post-transplant organ function (22). Another promising drug target is the apoptosis-related pathway. Transcriptomic studies have found that the expression of several apoptosis-related genes is significantly upregulated in DCD livers (29). By using apoptosis inhibitors such as caspase inhibitors, researchers successfully reduced cell apoptosis in DCD livers, thereby improving organ survival rates (30). These drug intervention strategies, guided by functional omics data, not only reveal the molecular mechanisms of DCD liver injury but also provide new directions for developing more effective treatments.

**Table 2:** Summary of therapeutic strategies for DCD liver grafts based on omics data.

Author,country,Publication year [Reference]	Omics platform	Detection content	Key Findings	Treatments	Main Effects
Eri H. Kobayashi, JP, 2016 (25)	Transcriptomics	1. Microarray analysis:Analyze gene expression data from bone marrow-derived macrophages (BMDMs). 2. ChIP-sequencing:Detection of Nrf2 binding sites in the genome 3. Real-time quantitative PCR analysis:Verify the expression level of a specific gene 4. Immunoblot analysis:Protein expression and activity were measured	Regulation of Nrf2 for antioxidant response in DCD conditions	Modulation of Nrf2 pathways	Enhanced graft resilience
Yimou Lin, CN, 2024 (2)	Proteomics, Metabolomics	1. Analysis of graft biopsies to identify differentially expressed proteins (DEPs) between EAD and non-EAD groups. 2. Analysis of paired perfusates to identify differentially expressed metabolites (DEMs) between EAD and non-EAD groups.	1. Identified 335 DEPs between EAD and non-EAD groups, significantly enriched in triglyceride and glycerophospholipid metabolism, neutrophil degranulation, and MET-related signaling pathway. 2. Found 193 DEMs in LC–MS-based platform and 94 DEMs in GC–MS-based platform, enriched in GPL metabolism, ABC transporters, and pentose and glucuronate interconversions.	1. Proteomics-Model: An integrated XGBoost machine learning model based on DEPs. 2. Integrated-Model: A predictive model using clinical parameters and perfusate metabolic products.	1. Showed high accuracy in distinguishing EAD cases from non-EAD cases. 2. Displayed superior predictive efficacy compared to existing models, with an AUC of 0. 915 in the training set and 0. 833 in the validation set. 3. Identified novel biomarkers in both grafts and perfusates that could be used to assess graft quality and provide new insights into the etiology of EAD.
Mamatha Bhat, CA, 2020 (26)	Metabolomics	1. Metabolite Analysis: Metabolite analysis was conducted using a Sciex 6600 Q-TOF high-resolution mass spectrometer. 2. Sample Processing: Rapidly frozen liver biopsy samples were analyzed using an LC/MS/MS high-resolution mass spectrometer. 3. Data Quantification: The abundance of metabolites was expressed as absolute quantification (ng/mg of tissue), obtained using a concentration curve for each metabolite.	NEsLP significantly improves mitochondrial metabolism in DCD liver grafts, including1. enhanced glycolysis2. fatty acid oxidation3. urea cycle function	Application of normothermic *ex situ* liver perfusion (NEsLP) during preservation	1. Improved mitochondrial function, 2. reduced ischemia-reperfusion injury, enhanced graft viability
Sandra V. Mateo, ES, 2024 (27)	Transcriptomics	1. Immunofluorescence Analysis: Used to detect the activation of inflammasome complexes in liver tissue, particularly identifying the activation of inflammasomes through ASC “specks. ” 2. Real-time Quantitative PCR (qRT-PCR): Employed to analyze the expression of genes related to the NLRP3 inflammasome. 3. Western Blot Analysis: Used to detect the expression of ASC and caspase-1 proteins in liver tissue. 4. Enzyme-linked Immunosorbent Assay (ELISA): Utilized to measure the concentration of IL-1β in liver tissue	Higher expression of inflammasome-related genes (NLRP3, ASC, Caspase-1, IL-1β, and IL-18) observed in DCD liver tissues before and after cold ischemia, associated with increased inflammatory response	Inhibition of inflammasome activation using specific inhibitors	1. Reduced inflammation, decreased cell death, 2. improved liver graft function

Nrf2, Nuclear Factor Erythroid 2-related Factor 2; ROS, Reactive Oxygen Species; RNS, Reactive Nitrogen Species; DCD, Donation after Circulatory Death; ATP, Adenosine Triphosphate; NEsLP, Neuroendocrine Longevity Protein; ASC, Apoptosis-associated speck-like protein.

Gene therapy, as an emerging treatment approach, has also shown great potential in protecting DCD livers. Through gene editing technologies like CRISPR-Cas9, scientists can precisely regulate the expression of key genes in DCD livers to mitigate IRI-induced damage (31). For example, researchers have used CRISPR-Cas9 technology to knock out specific genes responsible for inflammatory responses, such as NF-κB, in DCD livers, significantly reducing the release of inflammatory mediators and protecting hepatocytes (32). Additionally, gene transfer techniques have been used to enhance the antioxidant capacity of DCD livers. By introducing genes encoding antioxidant enzymes (such as superoxide dismutase [SOD] and glutathione peroxidase [GPX]) into donor liver cells, researchers successfully increased the liver’s antioxidant defenses and reduced oxidative stress damage caused by IRI (10). These gene therapy studies not only demonstrate the potential of gene regulation in protecting DCD livers but also lay the groundwork for future clinical applications. Metabolic regulation also plays a significant role in treatment, with metabolomic studies revealing the severity of metabolic disturbances in DCD livers during IRI, particularly in energy metabolism and lipid metabolism (33). These findings have prompted scientists to develop a series of metabolic regulation strategies to restore the normal metabolic functions of donor livers. For example, studies have shown that ATP depletion is severe during ischemia in DCD livers, and lactate accumulation leads to acidosis (31). Based on this, metabolic regulators such as pyruvate and succinate have been used in experimental studies, showing effects in restoring cellular energy balance and neutralizing acidosis, thereby improving post-transplant organ function (34). Additionally, lipid metabolism regulation has become an important research direction. Metabolomic analysis has found that lipid peroxidation is significantly enhanced in DCD livers, leading to cell membrane damage and functional decline (35). Researchers have successfully reduced the formation of peroxidized lipids and protected the integrity of hepatocyte membranes by using lipid peroxidation inhibitors, such as vitamin E and carotenoids (2). These metabolic regulation strategies provide new methods for improving the metabolic state of DCD livers and reducing IRI damage.

## 6 Emerging therapeutic approaches in experimental studies and clinical trials

Recent advancements in functional omics, including transcriptomics, proteomics, and metabolomics, have led to the identification of key molecular mechanisms underlying ischemia-reperfusion injury (IRI) in donation after circulatory death (DCD) liver grafts. These insights are paving the way for novel therapeutic strategies aimed at improving the quality of DCD organs and enhancing post-transplant outcomes (2).

Transcriptomic analysis has revealed that genes like HIF-1α, related to hypoxic adaptation, are upregulated during ischemia in DCD livers, suggesting potential targets for therapeutic modulation. Transcriptomic profiling has identified key inflammatory pathways involved in IRI, such as IL-6 and TNF-α, which could be targeted for anti-inflammatory interventions (36). For instance, inhibiting the overexpression of HIF-1α can reduce inflammation and cell death, improving organ viability. Additionally, anti-inflammatory agents targeting upregulated pro-inflammatory genes such as IL-6 and TNF-α have shown promise in mitigating the inflammatory response and protecting organ function. Recent studies also highlight the potential of regulating genes like Nrf2, which are involved in antioxidant responses, to improve the resilience of DCD grafts.

Proteomic analyses have identified reductions in mitochondrial function-related proteins, such as ATP synthase, which contribute to energy depletion and oxidative stress. Proteomic insights have highlighted mitochondrial dysfunction as a critical factor affecting graft viability and energy metabolism in DCD livers (37). Antioxidant therapies, including N-acetylcysteine (NAC) and vitamin E, have shown potential in reducing oxidative stress and protecting mitochondrial integrity. Moreover, caspase inhibitors targeting apoptosis-related proteins have been explored to mitigate cell death, further improving organ survival post-transplant. Additionally, proteomic data suggest that increasing the expression of mitochondrial protective proteins, such as SOD2, could further reduce oxidative damage and enhance graft quality.

Metabolomic profiling has highlighted significant metabolic disturbances, including disruptions in glycolysis and fatty acid oxidation. Metabolomic studies have identified mitochondrial dysfunction and disruptions in energy metabolism, which are key contributors to the severity of ischemia-reperfusion injury (12). Elevated lactate levels indicate energy depletion and metabolic acidosis, contributing to graft dysfunction. Metabolic interventions, such as pyruvate or succinate administration, have been effective in restoring energy homeostasis and improving post-transplant function. Furthermore, metabolomic analysis has shown that supplementation with certain amino acids, such as glutamine, can help maintain mitochondrial function and reduce IRI severity. Lipid peroxidation inhibitors like vitamin E and carotenoids also help prevent membrane damage, enhancing liver stability (4).

Integrating transcriptomic, proteomic, and metabolomic data provides a comprehensive understanding of IRI in DCD livers. A multi-target approach combining anti-inflammatory drugs, antioxidants, and metabolic regulators may offer a robust strategy to protect DCD livers. Machine perfusion, combined with these therapeutic agents, has also shown promise in maintaining organ viability and enhancing post-transplant recovery. Machine perfusion techniques have been shown to effectively reduce ischemia-reperfusion injury and improve DCD liver graft outcomes (38).

While these strategies hold great promise, challenges remain in translating findings into clinical practice. The integration of multi-omics data requires advanced bioinformatics tools and collaboration, and clinical trials are needed to evaluate the safety and efficacy of these therapies. Moving forward, personalized treatment approaches incorporating patient-specific omics profiles may become a cornerstone of precision medicine in organ transplantation, offering tailored strategies to improve outcomes for DCD liver graft recipients.

## 7 Limitations for omics study in DCD donors complexity of data

Functional omics involves generating vast datasets through high-throughput sequencing and multi-omics approaches, such as transcriptomics, proteomics, and metabolomics (39). This data is highly complex, necessitating advanced bioinformatics tools for accurate interpretation. Additionally, the heterogeneity among DCD donors—including differences in ischemia times, donor age, and comorbidities—further complicates data analysis (40).

## 8 Difficulties in Data Integration

Integrating transcriptomic, proteomic, and metabolomic data to create a comprehensive understanding of molecular changes in DCD organs is challenging. Discrepancies between mRNA levels and corresponding protein expression, which can result from post-transcriptional modifications or degradation, can lead to conflicting results and misinterpretations if not carefully handled (39, 41).

## 9 Technical limitations

High-throughput sequencing technologies are powerful but costly, and they require specialized equipment and expertise (42). This presents a significant barrier for institutions with limited resources to engage in functional omics research. Moreover, preserving high-quality DCD liver samples remains challenging because ischemia-reperfusion injury often affects tissue integrity, thus compromising the reliability of the research results (43).

## 10 Reproducibility issues

Reproducibility is a major concern in omics research. Variations in sample processing, sequencing workflows, and data analysis techniques can yield inconsistent results, making it difficult to derive definitive conclusions. Therefore, the development of standardized research protocols and more robust bioinformatics tools is critical for ensuring the reliability and reproducibility of findings.

## 11 Ethical considerations

As the use of DCD organs becomes more prevalent in clinical transplantation, implementing omics-based assessment technologies raises important ethical and logistical considerations. One key concern is the handling of genetic data from functional omics research, which involves issues of privacy, informed consent, and potential genetic discrimination (44). It is crucial to establish a robust regulatory framework that ensures the responsible use of genetic information while protecting donor rights. Additionally, gene-editing technologies such as CRISPR-Cas9, still in their early stages, face strict ethical scrutiny regarding their application in human genetics. From a logistical perspective, while omics technologies hold great promise for improving organ quality assessment, their clinical implementation requires high-end equipment, specialized expertise, and significant financial investment, which may not be available in all transplant centers. The time-sensitive nature of DCD organ transplantation also presents challenges, as omics data analysis can be time-consuming, and the window for organ preservation is limited. Therefore, integrating these technologies into clinical practice will require careful consideration of both ethical principles and practical feasibility to ensure fair, safe, and effective use.

## 12 Future directions and prospects in functional omics of DCD donors

The field of functional omics in heart-dead donor (DCD) liver research holds significant promise for future advancements.

Firstly, progress in data integration and analysis will be crucial. To manage the complexity of functional omics data, future research must focus on developing advanced bioinformatics tools capable of integrating multi-omics data into actionable insights. Artificial intelligence (AI) and machine learning (ML) algorithms have shown significant potential for handling large and complex datasets and are expected to revolutionize the interpretation of functional omics data, enabling more precise biomarker discovery and personalized treatment strategies (45).

Secondly, innovative therapeutic approaches will become a focal point of future research. Omics-based interventions may be combined with emerging technologies, such as regenerative medicine and organ-on-a-chip models, providing new insights into DCD liver injury mechanisms and identifying new therapeutic targets. For example, combining gene editing and stem cell therapy may enhance liver regeneration and improve outcomes for DCD liver transplantation (46, 47).

Thirdly, global collaboration and standardization are vital. Addressing the technical and ethical challenges requires increased cooperation among global research institutions, industry partners, and regulatory agencies. Establishing standardized processes for generating, analyzing, and reporting omics data will improve the reproducibility and comparability of findings across different settings. Additionally, international guidelines on the ethical use of genetic data and gene editing in transplantation will ensure that research advances are responsibly and equitably translated into clinical practice (48, 49).

In terms of clinical implementation, as omics technologies become more integrated into clinical practice, patient-centered care will grow in importance. This involves ensuring that patients benefit from the latest advancements while addressing the ethical, social, and psychological impacts of omic medicine. Patient education and engagement will be key to the successful implementation of omics-based therapies (50, 51).

Looking ahead, several key research areas will shape the development of functional omics in DCD donors. Firstly, epigenomics plays a significant role during ischemia and reperfusion, and understanding these changes could reveal new targets for therapeutic intervention to improve long-term transplant outcomes. Secondly, organoid models and bioengineering approaches provide valuable platforms for studying DCD organ responses and testing new therapies. Combining organoid technology with functional omics may accelerate the discovery of new treatments and improve our understanding of DCD molecular mechanisms.

Lastly, ongoing efforts to improve the integration and interpretation of multi-omics data will drive the field forward. Collaboration among researchers, omics experts, bioinformaticians, and clinical medicine specialists will be key to translating these discoveries into practical solutions. Overall, the advancement of functional omics in DCD donors is promising. A deeper understanding of the molecular and cellular mechanisms of DCD organ injury has the potential to revolutionize organ preservation, donor selection, and post-transplant care. However, realizing this potential will require continued research, technological innovation, and addressing the ethical and policy challenges of omic data use. As research continues, functional omics is expected to play an increasingly crucial role in the future of DCD organ transplantation.

## 13 Conclusions

In this study, we systematically explored the molecular mechanisms underlying the inferior quality of liver grafts from donation after circulatory death (DCD) using multi-omics approaches. Our findings highlighted key biological pathways, including oxidative stress, inflammation, and mitochondrial dysfunction, that contribute to the deterioration of DCD liver quality. Omics research plays a crucial role in understanding these complex mechanisms, offering valuable insights into potential biomarkers and therapeutic targets for improving organ function. Looking forward, integrating multi-omics data, developing targeted interventions, and enhancing preservation techniques will be essential for improving the quality of DCD liver grafts, ultimately enhancing transplant success rates and patient outcomes.
