# Correction: Current understanding on inferior quality of liver grafts by donation after circulatory death based on multi-omics data

**DOI:** 10.3389/fimmu.2025.1633195

**Published:** 2025-06-12

**Authors:** Yifeng Zhou, Ting Que, Lu Yu, Shuping Que, Jun Xu, Zhengtao Liu

**Affiliations:** ^1^ Key Laboratory of Artificial Organs and Computational Medicine in Zhejiang Province, Shulan International Medical College, Zhejiang Shuren University, Hangzhou, China; ^2^ Birth Defects Prevention and Control Institute, Maternal and Child Health Hospital of Guangxi Zhuang Autonomous Region, Nanning, China; ^3^ School of Medicine, Zhejiang Chinese Medical University, Hangzhou, China; ^4^ Shulan (Hangzhou) Hospital, Hangzhou, China; ^5^ Ya-er-zhuang Clinics, Hangzhou, China; ^6^ Division of Hepatobiliary and Pancreatic Surgery, Department of Surgery, First Affiliated Hospital, School of Medicine, Zhejiang University, Hangzhou, China; ^7^ NHC Key Laboratory of Combined Multi-organ Transplantation, Key Laboratory of the Diagnosis and Treatment of Organ Transplantation, School of Medicine, Chinese Academy of Medical Sciences, First Affiliated Hospital, Zhejiang University, Hangzhou, China; ^8^ Key Laboratory of Organ Transplantation, First Affiliated Hospital, School of Medicine, Zhejiang University, Hangzhou, China

**Keywords:** donation after circulatory death, liver transplantation, multi-omics, ischemia-reperfusion injury, oxidative stress, inflammatory response

In the published article, there was an error in the author list. The author Ting Que should have been listed as a co-first author. The corrected author list appears below:

“Yifeng Zhou*, Ting Que*, Lu Yu, Shuping Que, Jun Xu, and Zhengtao Liu.”

(*Co-first authors).

The original article has been updated.

